# Contrast-enhanced mammography BI-RADS: a case-based approach to radiology reporting

**DOI:** 10.1186/s13244-024-01612-z

**Published:** 2024-02-08

**Authors:** Luca Nicosia, Ottavia Battaglia, Massimo Venturini, Federico Fontana, Manuela Minenna, Aurora Pesenti, Diana Budascu, Filippo Pesapane, Anna Carla Bozzini, Maria Pizzamiglio, Lorenza Meneghetti, Antuono Latronico, Giulia Signorelli, Luciano Mariano, Enrico Cassano

**Affiliations:** 1 Breast Imaging Division, Radiology Department, IEO European Institute of Oncology IRCCS, 20141 Milan, Italy; 2 Postgraduation School of Diagnostic and Interventional Radiology, University of Milan, Via Festa del Perdono 7, 20122 Milan, Italy; 3 Diagnostic and Interventional Radiology Department, Circolo Hospital, ASST Sette Laghi, 21100 Varese, Italy; 4 School of Medicine and Surgery, Insubria University, 21100 Varese, Italy; 5 Department of Radiology, IEO European Institute of Oncology IRCCS, 20141 Milan, Italy; 6 Radiology Department, Università degli Studi di Torino, 10129 Turin, Italy

**Keywords:** Mammography, Quality data reporting, Contrast agent, Breast diseases

## Abstract

Contrast-enhanced mammography (CEM) is a relatively recent diagnostic technique increasingly being utilized in clinical practice. Until recently, there was a lack of standardized reporting for CEM findings. However, this has changed with the publication of a supplement in the Breast Imaging Reporting and Data System (BI-RADS). A comprehensive understanding of CEM is essential for further enhancing its role in both screening and managing patients with breast malignancies. CEM can also be beneficial for problem-solving, improving the management of uncertain breast findings. Practitioners in this field should become more cognizant of how and when to employ this technique and interpret the various CEM findings. This paper aims to outline the key findings in the updated version of the BI-RADS specifically dedicated to CEM. Additionally, it will present some clinical cases commonly encountered in clinical practice.

**Critical relevance statement** Standardized reporting and a thorough understanding of CEM findings are pivotal for advancing the role of CEM in screening and managing breast cancer patients. This standardization contributes significantly to integrating CEM as an essential component of daily clinical practice.

**Key points** • A complete knowledge and understanding of the findings outlined in the new BI-RADS CEM are necessary for accurate reporting.

• BI-RADS CEM supplement is intuitive and practical to use.

• Standardization of the CEM findings enables more accurate patient management.

**Graphical Abstract:**

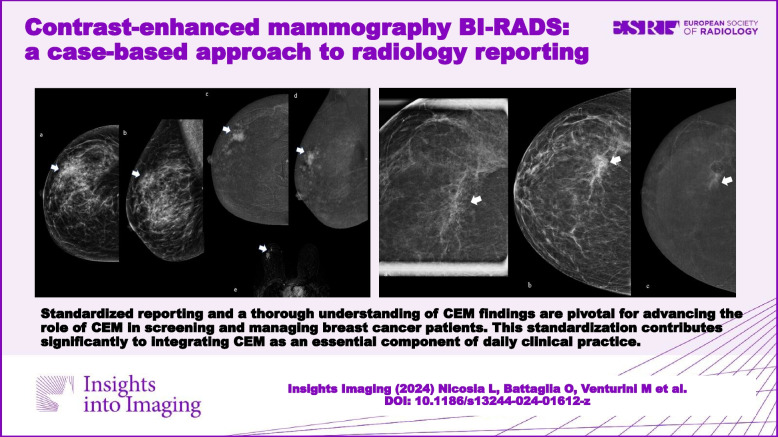

## Introduction

Mammography has been increasingly utilized for annual screening and breast cancer patient follow-up since the early 1970s. However, the initial absence of a standardized mammographic report posed a significant limitation to the effective communication of results between radiologists, patients, and other physicians, leading to suboptimal patient management [1, 2]. BI-RADS, a classification proposed by the American College of Radiology (ACR), was introduced to address this issue by employing a specific lexicon to optimize and standardize radiological reports. The first BI-RADS classification was introduced in 1993, focusing on the mammographic report, the lexicon for mammographic imaging findings, and the final assessment category with recommendations for management. The BI-RADS classification aims to eliminate ambiguity, facilitate better data collection, and enhance communication with patients and referring physicians [3, 4].

This system has evolved over the years, with the latest edition being the 5th, released in 2013 [4], which includes dedicated sections on ultrasound (US) and magnetic resonance imaging (MRI) [5, 6]. Meanwhile, the sixth edition is currently under development. In 2022, a new supplemental section on CEM was released, acknowledging its increased usage and physicians’ experience since its initial Food and Drug Administration (FDA) approval in 2011 [7].

Numerous studies have confirmed CEM’s utility and diagnostic performance in clinical settings [8]. However, until recently, a major drawback of this method was the lack of standardization in reporting the findings that radiologists may encounter [9–12].

This article offers an overview of the BI-RADS CEM lexicon and demonstrates a structured reporting approach through illustrative cases, aiding in BI-RADS reporting.

### CEM technique

CEM is a relatively recent and promising diagnostic technique that combines information obtained from a standard digital mammography (DM) with that obtained after contrast-agent administration. CEM involves dual-energy exposure in conjunction with the injection of an iodinated contrast agent, employing principles similar to breast MRI, particularly exploiting tumor angiogenesis [13].

#### Contrast agent injection

An iodinated contrast agent is injected at a dose of 1.5 mL/kg before acquiring two images in each projection (usually cranio-caudal and medio-lateral-oblique) on each side. The contrast-agent allows visualization of blood vessels and lesions in breast tissue.

#### Dual-energy exposure

Following contrast-agent injection, approximately 2 min are generally required for the agent to circulate and be absorbed by the breast tissue. Dual-energy imaging involves taking X-ray images at two different energy levels: low energy (LE) and high energy (HE) [13].

- LE exposure: This utilizes the same X-ray energy spectrum as a standard full-field DM with a peak kilovoltage (KVp) of around 30 KVp. LE image resembles a conventional DM with a comparable diagnostic capacity [14].
- HE exposure: Is typically performed at around 45 KVp, exploiting the higher K-edge of iodine. This energy level allows iodine in the contrast agent to stand out, providing information about contrast medium uptake and highlighting areas with increased blood supply, such as tumors. The latter image is not used for diagnostic purposes [13].

#### Image processing

Acquired images are then processed using a dual-energy-weighted logarithmic subtraction technique, resulting in two sets of images:

- LE image: This image resembles a standard DM and provides structural information about breast tissue.
- Recombined (RC) image: Is generated by combining LE and HE images. In this image, areas of contrast medium uptake appear enhanced, aiding in identifying areas with increased blood flow [13].

The CEM dual-energy approach allows for improved contrast and visualization of lesions with enhanced vascularity (angiogenesis). It is particularly beneficial in cases where traditional DM might not provide sufficient information, such as for women with dense breast tissue: many lesions having a subtle appearance on standard MD, especially in dense breasts, will manifest more prominently [8]. CEM provides essential details about enhancing a lesion while retaining valuable information from DM, such as the morphology of microcalcifications, thanks to the LE image.

According to one of the most comprehensive meta-analyses published by Cozzi et al. [8], CEM showed a sensitivity of 95% (CI: 92, 97) in detecting cancers in patients with dense breasts compared to the very low sensitivity (around 60%) demonstrated by DM alone [9]. Some recent papers have been published on specificity. CEM outperforms DM in specificity: values are close to 80% [8]. Compared with MRI, CEM also has some advantages; it is faster, cheaper, and allows an assessment of the morphology of microcalcifications [10].

CEM may be employed in different settings, with important indications, among the main ones being as follows:

- Locoregional staging of new breast cancer (especially in dense breasts): CEM demonstrates similar efficacy as MRI in evaluating tumor extension [15].
- Problem-solving concerning breast findings of difficult interpretation by conventional imaging, such as DM or US (including microcalcifications) [16, 17]: CEM appears to play an essential role in optimizing the workflow of biopsies required by radiologists and in highlighting breast findings that could be easily missed or underestimated.
- Evaluation of response to neoadjuvant therapy [18]: CEM appears to have a diagnostic performance comparable to MRIs in assessing response to therapy. Compared with MRI, it offers the advantage of lower cost and faster execution.
- Screening of high-risk patients with dense breasts is beginning to be considered: CEM appears to demonstrate a superior sensitivity in tumor detection to classical DM and not inferior to tomosynthesis [8, 9]. Furthermore, compared with tomosynthesis examinations, preliminary results show that in CEM, the breast is not exposed to a higher average glandular dose [19].
- Surveillance in patients with a prior history of breast cancer: CEM proves to be an excellent tool for use in the surveillance of oncological patients [20]. According to Elder et al. [20], when compared to DM as a surveillance modality, CEM has higher sensitivity and can identify additional malignant lesions that are clinically significant. It seems particularly useful in the study of surgical scars and in evaluating findings whose interpretation is ambiguous on DM and US [20].

### BI-RADS CEM overview

BI-RADS CEM lexicon integrates terminology used in the fifth edition for DM and MRI descriptions.

### Breast tissue and background parenchymal enhancement (BPE)

Breast tissue composition is assessed on LE, utilizing the same lexicon as conventional DM [4], categorizing it into four main groups (almost entirely fatty, scattered areas of glandular density, heterogeneously dense, and extremely dense).

BPE is described using the lexicon from breast MRI, visually estimating the enhancement level of glandular tissue after contrast-agent administration. BPE is divided into four categories (minimal, mild, moderate, and marked) and can be symmetric or asymmetric between the two breasts.

### Findings

Three major categories can be distinguished: (1) Findings on LE images only, (2) enhancement on RC images only, and (3) findings seen on LE images with enhancement on RC images associated.

If an abnormality has suspicious features on LE images but not on RC ones, it should still be considered suspicious (e.g., calcifications) [16].

1. ***Findings on LE images only***
  *Masses*: A mass is a 3-D space-occupying lesion that may be recognized on LE images with or without a corresponding abnormal enhancement on RC. Descriptors include shape (oval, round, irregular), margins (circumscribed, obscured, micro-lobulated, indistinct, spiculated), and density (high density, equal density, low density, and fat containing).
  *Calcifications*: Two main categories are typically benign and suspicious morphology. According to recent studies, the absence of contrast enhancement of microcalcifications in RC image is not necessarily associated with the absence of pathology [16]; therefore, microcalcifications with suspicious morphology in LE image should lead to biopsy.
  Architectural distortions
  *Asymmetries*: Including focal, global, and developing asymmetries, should be evaluated and managed as on any DM, and whether they enhance or not.
2. ***Findings on RC images only***
  Similar to MRI, enhancement on an RC image is described as a mass, non-mass, or enhancing asymmetry per the CEM lexicon.
  *Masses*: Shape descriptors include oval, round, and irregular, with margin categories circumscribed and non-circumscribed. Internal enhancement may be homogeneous, heterogeneous, or rim enhancement.
  *Non-masses*: Enhancement without a space-occupying effect is classified as a non-mass enhancement. Descriptors include distribution (focal, linear, segmental, regional, multiple regions, or diffuse) and internal enhancement (homogeneous, heterogeneous, clumped). “Clustered ring enhancement,” used in MRI, is not included.
  *Lesion conspicuity*: A new descriptor defined as the enhancement degree relative to the background, categorized as low, moderate, or high. Lesion conspicuity is related to the malignancy of lesions and the receptor profile of malignant breast cancer [12].
3. ***Associated features***

Useful to further characterize main findings and corroborate suspicion of malignancy, including the following:

- *Nipple retraction*
- *Nipple invasion*
- *Skin retraction*
- *Skin thickening*
- *Skin invasion*
- *Trabecular thickening*
- *Axillary adenopathy*

### Assessment and management recommendations

All CEM examinations should include assessment and management recommendations, categorized from 0 to 6, and the report should be systematically structured:

- *Category 0 (incomplete)*: Recall for additional imaging and/or comparison with prior examination
- *Category 1 (negative)*: Routine screening
- *Category 2 (benign)*: Routine screening
- *Category 3 (≤ 2% likelihood of malignancy)*: Short interval (6 months) follow-up
- *Category 4 (≥ 2% but < 95% chance of malignancy)*: Tissue diagnosis
- *Category 5 (≥ 95% chance of malignancy)*: Tissue diagnosis
- *Category 6 (known biopsy-proven malignancy)*: Surgical excision when clinically appropriate

### Structured report

The report should be systematically structured:

1. *Indication for the examination*
2. *CEM technique*
3. *Comparison to previous examinations*
4. *Succinct description of overall breast composition*
5. *Clear description of any important finding*
6. *Assessment*
7. *Management*

### Clinical examples of a structured report according to new BI-RADS

**Fig. 1:**
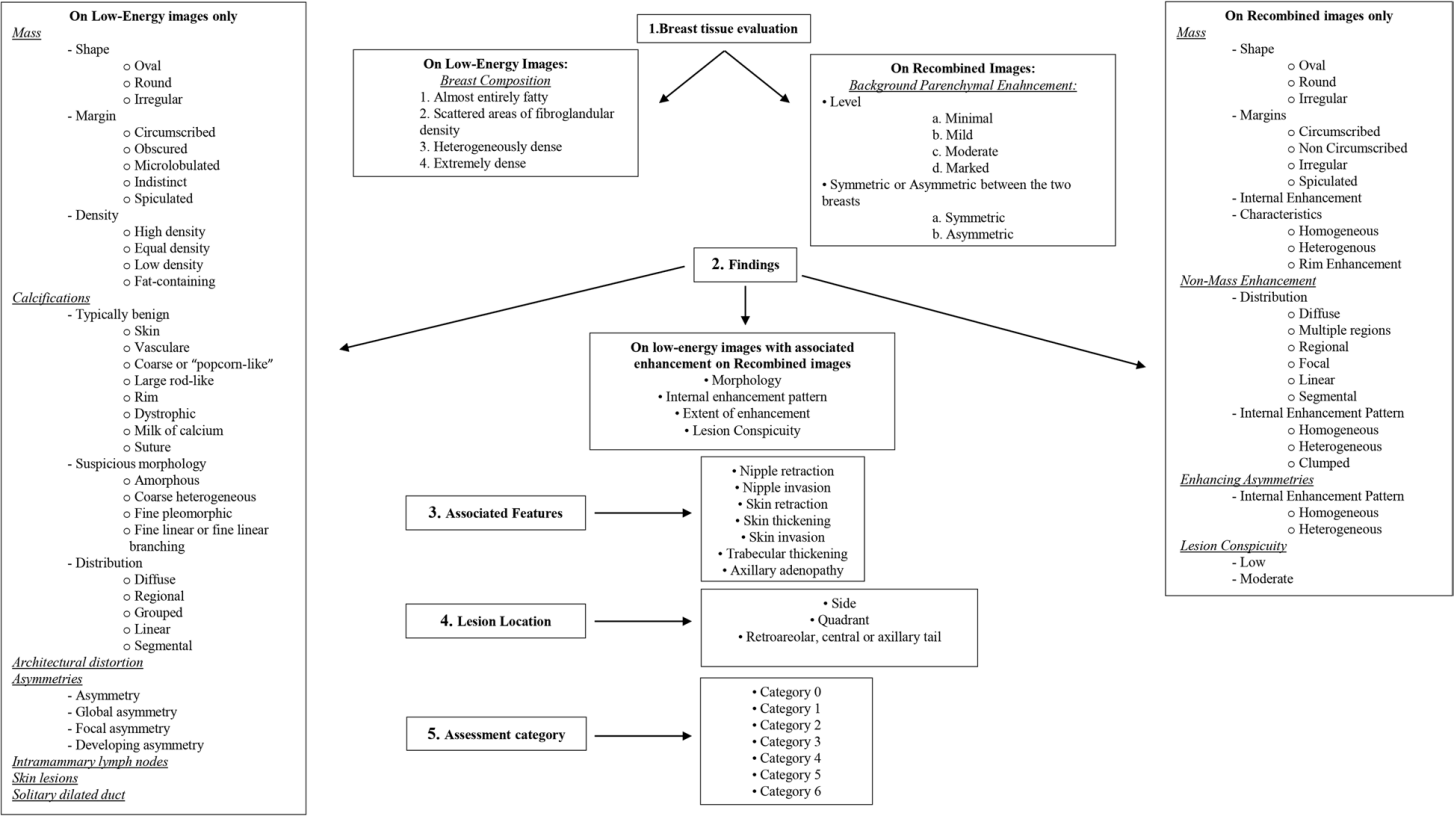
A summary description of the new features of BI-RADS CEM

The provided text describes clinical cases illustrating the application of structured reporting in alignment with the new BI-RADS. The details presented in these cases are based on Fig. 1, which outlines the summary characteristics of the new BI-RADS CEM and the “Structured report” section. Analyzing these clinical cases serves as a foundation for constructing a structured report. Adhering to the parameters of the new BI-RADS, reporting within this framework facilitates the creation of an organized description, proving beneficial to both the radiologist and the patient.

#### Case 1: Invasive ductal carcinoma manifesting as non-mass enhancement

**Fig. 2:**
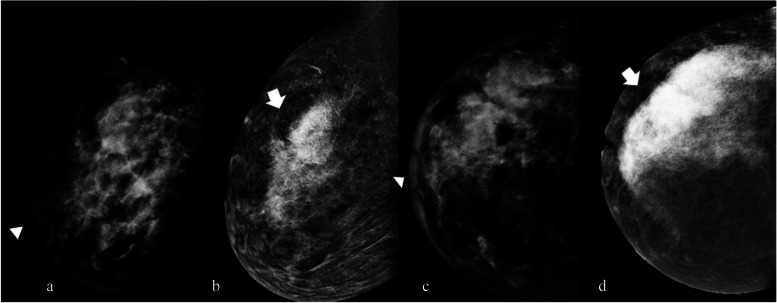
Invasive ductal carcinoma manifesting as non-mass enhancement in the RC image of CEM

A 40-year-old woman sought clinical attention due to breast pain and swelling with skin thickening associated. She underwent CEM before the biopsy (Fig. 2).

##### Structured report example

1. *Indication*: Right breast pain and swelling with skin thickening associated
2. *Technique*: Examination conducted after intravenous administration of iodine-based contrast medium (1.5 mL/kg). Two minutes after injection, DM projections (cranio-caudal and medio-lateral-oblique) were acquired at both LE (26–32 kVp) and HE (45–49 kVp). An RC image was then reconstructed to highlight any contrast medium uptake.
3. *Observations are as follows:*

- *Breast composition* (LE Fig. 1a and c): Heterogeneously dense (ACR C)
- *BPE (RC)*: Minimal (Fig. 2b and d)
- *Findings*
  • *LE image* (Fig. 1a and c): No specific breast changes are evident. Associated features are as follows: skin thickening (arrowhead, Fig. 1a and c).
  • *RC image* is as follows (Fig. 1b and d):
- Right non-mass enhancement
- Location
- Distribution: multiple regions (maximum extension of 10 cm)
- Pattern: heterogeneous
- Lesion conspicuity: High

4. *Assessment category*: BI-RADS 5
5. *Management*: A second look US was recommended to confirm any echo structural changes. Where non-mass enhancement was observed, an area of parenchymal distortion was highlighted, which was biopsied with US guidance, leading to a diagnosis of invasive ductal carcinoma. The patient subsequently underwent mastectomy with axillary dissection.

*Tips*: It is crucial to consistently consider associated features, such as skin thickening, which are frequently disregarded in the comprehensive assessment of DM. Having the radiologist present during the CEM examination to perform a clinical assessment afterwards would be beneficial. For instance, specific skin formations, such as nevi, are accentuated with CEM, and any uncertainties can be addressed effectively through a single clinical visit.

#### Case 2: Invasive ductal carcinoma manifesting as mass enhancement

**Fig. 3:**
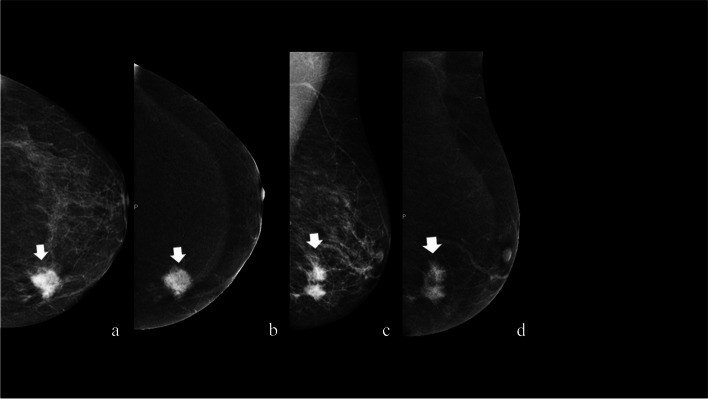
Invasive ductal carcinoma manifesting as a mass enhancement on CEM (typical presentation)

A 71-year-old female with a significant family history of breast cancer underwent CEM as a screening test, being classified as a high-risk patient (Fig. 3).

##### Structured report example

1. *Indication*: Screening examination in high-risk patient
2. *Technique*: Examination conducted after intravenous administration of iodine-based contrast medium (1.5 mL/kg). Two minutes after injection, DM projections (cranio-caudal and medio-lateral-oblique) were acquired at both LE (26–32 kVp) and HE (45–49 kVp). An RC image was then reconstructed to highlight any contrast medium uptake.
3. *Observations are as follows:*
  • *Breast composition* (LE): Scattered area of fibroglandular density (ACR: B)
  • *BPE* (RC): Minimal
  • *Findings*

*LE image* is as follows (Fig. 1a and c):

- Left mass lesion (maximum extension of 15 mm)
- Shape: Irregular
- Margins: Spiculated
- Density: High density
- Calcifications: No calcifications
- Associated features: No

*RC image* is as follows:

- Left mass enhancement (maximum extension of 15 mm)
- Location: Lower inner quadrant
- Shape: Irregular
- Margins: Spiculated
- Pattern: Heterogeneous
- Lesion conspicuity: High

4. *Assessment category*: BI-RADS 5

5. *Management*: A second look US was recommended to confirm any echo structural changes. Where mass enhancement was observed, a nodule with suspicious US characteristics (irregular margins, inhomogeneous, vascularized at color Doppler) was highlighted, which was biopsied with US guidance, leading to an invasive ductal carcinoma diagnosis. This is a typical presentation of invasive ductal carcinoma on CEM [11, 17]. The patient subsequently underwent mastectomy with sentinel lymph node biopsy.

*Tips*: CEM can serve as a viable screening choice for high-risk patients with dense breasts. The average glandular dose (AGD) does not exceed that of DM conducted with tomosynthesis, even when utilizing a single tomosynthesis projection [19].

#### Case 3: Invasive lobular carcinoma manifesting as non-mass enhancement

**Fig. 4:**
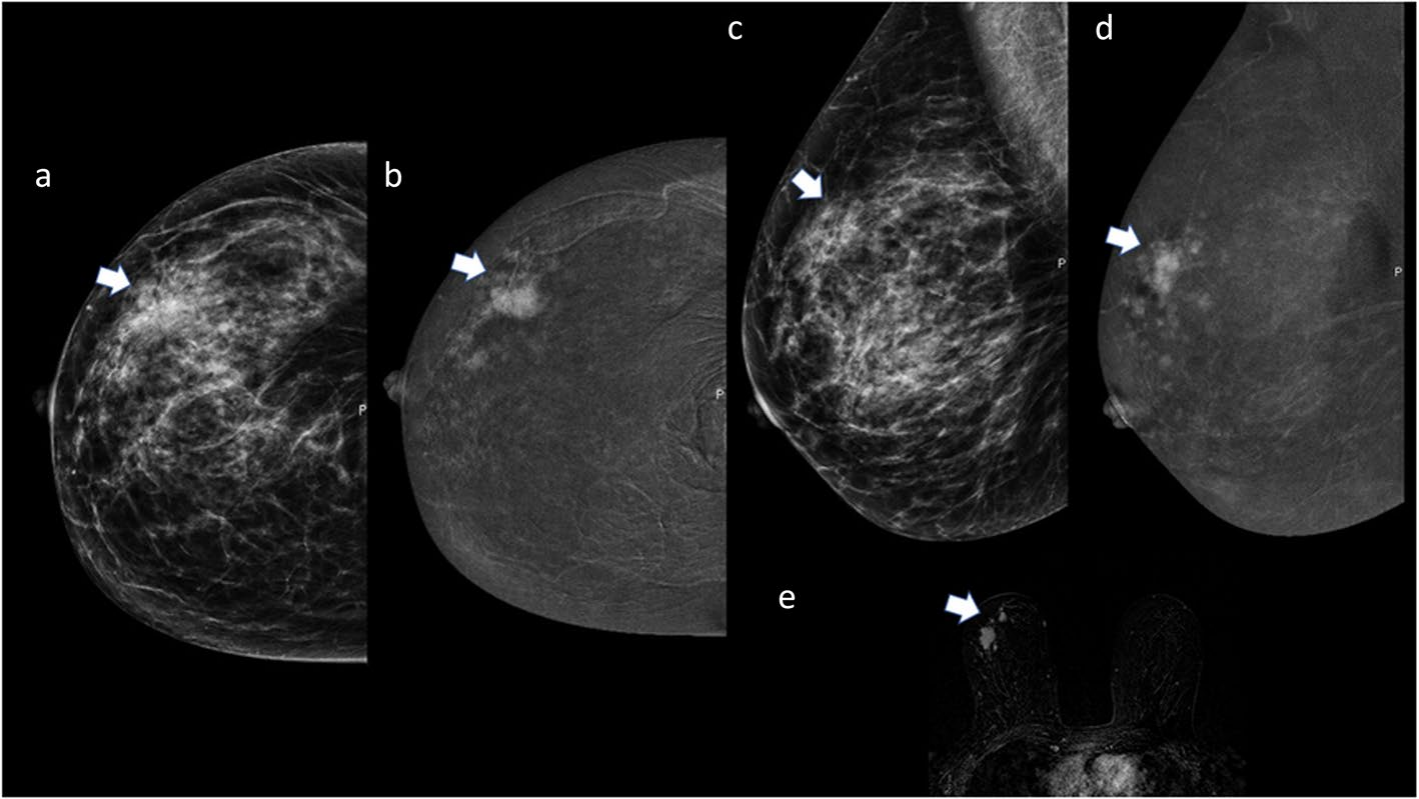
Typical presentation of invasive lobular carcinoma on CEM and comparison with breast MRI

In a 46-year-old woman with a BRCA2 gene mutation, a suspicious US finding was detected, and a core biopsy was performed with an infiltrative lobular carcinoma diagnosis. CEM and MRI were performed for preoperative staging (Fig. 4).

##### Structured report example

1. *Indication*: Staging in a patient with lobular neoplasm diagnosis
2. *Technique*: Examination conducted after intravenous administration of iodine-based contrast medium (1.5 mL/kg). Two minutes after injection, DM projections (cranio-caudal and medio-lateral-oblique) were acquired at both LE (26–32 kVp) and HE (45–49 kVp). RC image was then reconstructed to highlight any contrast medium uptake.
3. Observations
  • *Breast composition* (LE): Heterogeneously dense (ACR: C)
  • *BPE* (RC): Mild
  • *Findings*

- *LE image* is as follows (Fig. 4a and c):
  - ◦ Right architectural distortion (arrow)
  - ◦ No suspicious calcifications
  - ◦ Associated features: no
  - *◦ RC image* (Fig. 4b and c) is as follows:
  - ◦ Multifocal mass enhancement (arrow) with a maximum extension of 45 mm
  - ◦ Location: Lower inner quadrant
  - ◦ Shape: Irregular
  - ◦ Margins: Non-circumscribed
  - ◦ Pattern: Heterogeneous
  - ◦ Lesion conspicuity: High

4. Assessment category: BI-RADS 5
5. *Management*: The examination suggested a rather extensive multicentric tumor. A mastectomy was scheduled.
  A similar lesion was observed on MRI subtracted T1 image (Fig. 4e), where we can observe a mass enhancement with CEM-like characteristics.
  Recent publications [21, 22] have demonstrated a similar diagnostic performance between CEM and MRI in the management of lobular carcinoma. According to the limited literature on CEM and lobular neoplasms, lobular lesions often present as a multifocal mass enhancement on CEM, as illustrated in our case [21, 22].

*Tips*: CEM can be an alternative diagnostic tool to MRI, especially when MRI availability is limited or for patients experiencing claustrophobia. This is particularly relevant in the staging of lobular carcinoma. According to initial studies on the subject [21, 22], CEM demonstrates excellent performance in assessing the disease’s accurate extent and multicentric/focal nature. These preliminary studies [22] reveal no significant differences between the postsurgical pathological size of lesions and the size calculated through preoperative CEM and MRI. Consequently, CEM can offer valuable insights for surgical planning in patients with lobular neoplasia.

#### Case 4: Management of microcalcifications

**Fig. 5:**
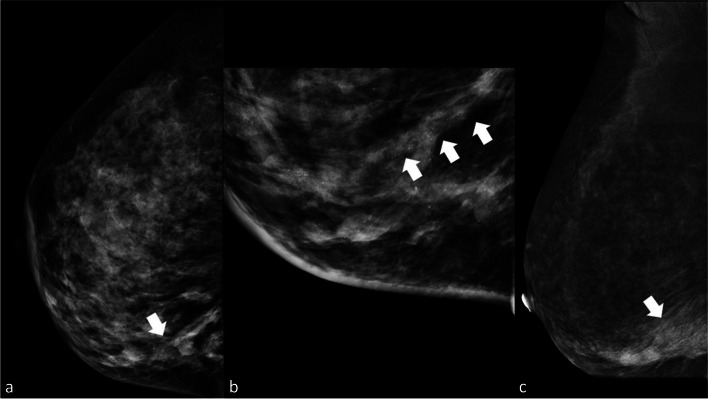
Utility of CEM in the management of microcalcifications

A 60-year-old asymptomatic woman underwent a screening DM, and amorphous microcalcifications were found in the inner quadrants of the right breast (Fig. 5). CEM could be used as a problem-solving technique (confirm or exclude a stereotactic biopsy).

##### Structured report example

1. *Indication*: Problem-solving in patient with indeterminate microcalcifications diagnosis
2. *Technique*: Examination conducted after intravenous administration of iodine-based contrast medium (1.5 mL/kg). Two minutes after injection, DM projections (cranio-caudal and medio-lateral-oblique) were acquired at both LE (26–32 kVp) and HE (45–49 kVp). RC image was then reconstructed to highlight any contrast medium uptake.
3. *Observations*
  • *Breast composition* (LE Fig. 1a and c): Heterogeneously dense (ACR C)
  • *BPE* (RC): Moderate (Fig. 5c)
  • *Findings*

- *LE image* (Fig. 5a): Coarse and heterogeneous microcalcifications extended over 5 cm in the right breast.

- ◦ Associated features: No

- *RC image is as follows* (Fig. 1b and d):

- ◦ Non-mass enhancement (same extension of microcalcifications)
- ◦ Distribution: Regional
- ◦ Pattern: Heterogeneous
- ◦ Lesion conspicuity: High
- ◦ Location: Inner quadrant

4. *Assessment category*: BI-RADS 4
5. *Management*: A stereotactic biopsy is required.

LE image on CEM (Fig. 5b and c) aligns with classical DM, as previously evidenced in the literature [14]. Microcalcifications pose a complex challenge: a biopsy referral is warranted if their appearance on RC images corresponds with contrast enhancement. However, if microcalcifications exhibit a suspicious morphology on LE images without a correlated contrast enhancement on RC images, histological verification is still recommended [16].

CEM proves particularly valuable in cases where microcalcifications display a morphology that is not distinctly suspicious, introducing potential management controversies.

The presence of enhanced microcalcifications often indicates the existence of breast pathology [16]; for instance, the histological result of the previously described case revealed a high-grade carcinoma in situ.

*Tips*: The level of suspicion regarding breast microcalcifications should be consistently assessed on LE image. Pathological microcalcifications may not necessarily exhibit enhancement. Nevertheless, the presence of enhancing microcalcifications increases the likelihood of malignancy. In instances where there is enhancement of microcalcifications that are not particularly suspicious, a biopsy should be deemed obligatory [16].

#### Case 5: Management of B3 high-risk lesions

**Fig. 6:**
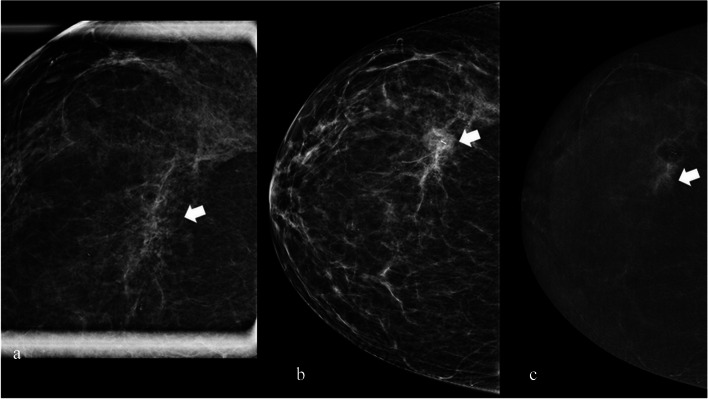
Utility of CEM in the management of B3 lesions

A 46-year-old woman underwent a screening DM, and suspicious tiny microcalcifications in the outer quadrants of the right breast were found (Fig. 6a; arrow: magnification of microcalcifications). A stereotactic biopsy was requested with an atypical ductal hyperplasia (B3 lesion) diagnosis and a few residual post-biopsy microcalcifications. After the biopsy, CEM was performed to evaluate the possibility of avoiding surgery.

##### Structured report example

1. *Indication*: Post-biopsy management of B3 lesion.
2. *Technique*: Examination conducted after intravenous administration of iodine-based contrast medium (1.5 mL/kg). Two minutes after injection, DM projections (cranio-caudal and medio-lateral-oblique) were acquired at both LE (26–32 kVp) and HE (45–49 kVp). An RC image was then reconstructed to highlight any contrast medium uptake.
3. *Observations*
  • *Breast composition* (LE Fig. 6a and b): Scattered areas of fibroglandular density (ACR B)
  • *BPE* (RC): Minimal (Fig. 6c)
  • *Findings*

- *LE image* (Fig. 6a): Fine and pleomorphic microcalcifications with a maximum extension of 20 mm in the right breast
- Associated features: No
- *RC image* (Fig. 6c)
  - Non-mass enhancement (same extension of microcalcifications)
  - Location: Central quadrant
- Distribution: Linear
  - Pattern: Heterogeneous
  - Lesion conspicuity: Moderate
  - Note: A mild rim enhancement around the post-biopsy marker was detected as a biopsy outcome.

4. *Assessment category*: BI-RADS 4
5. *Management*: The patient was scheduled for surgery based on suspicions of residual disease within the biopsy area despite the B3 histological result of the biopsy (atypical ductal hyperplasia), which could have allowed for active surveillance [23, 24].

These findings indicated a potential persistence of the disease. Subsequently, the patient underwent conservative peri-biopsy surgery, revealing a few foci of low-grade carcinoma in situ.

The role of CEM in B3 lesion management, in situations where the necessity of surgical intervention is still debated, as in the case of atypical ductal hyperplasia [23, 24], may be significant: the presence of peri-biopsy enhancement could serve as an indication for surgery [25].

*Tips*: Post-biopsy CEM should ideally be conducted at least 2 weeks after biopsy [26] to mitigate the impact of the procedure [26]. Even at this interval, rim enhancement is frequently observable as a subtle, circular enhancement surrounding the biopsied area. This form of enhancement is also commonly observed in cases of inflammatory cysts.

#### Case 6: Management of benign lesions

**Fig. 7:**
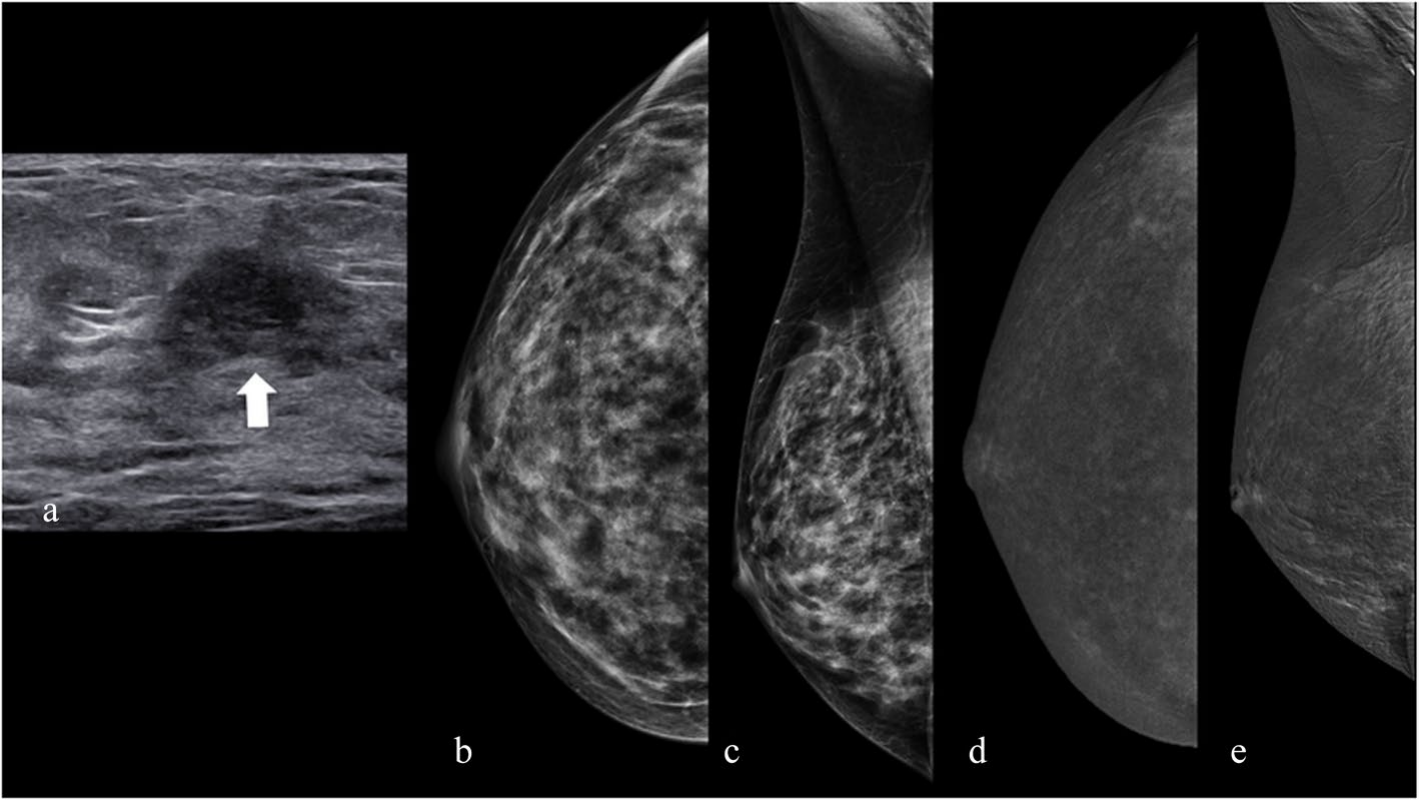
The absence of any suspicious findings is noted in both the RC and LE images corresponding to the location in the breast where a nodule was detected. The nodule presented challenges in interpretation through breast ultrasound

A 46-year-old woman underwent a screening US, and a mass of difficult interpretation was detected (Fig. 7a) with shadowy margins, although still well-defined, slightly inhomogeneous, and with poor vascularization on color Doppler. CEM was performed as a problem-solving technique.

##### Structured report example

1. *Indication*: Problem-solving of nodules with difficult interpretation
2. *Technique*: Examination conducted after intravenous administration of iodine-based contrast medium (1.5 mL/kg). Two minutes after injection, DM projections (cranio-caudal and medio-lateral-oblique) were acquired at both LE (26–32 kVp) and HE (45–49 kVp). An RC image was then reconstructed to highlight any contrast medium uptake.
3. Observations
  *Breast composition* (LE Fig. 7b–c): Heterogeneously dense (ACR C)
  *BPE* (RC): Moderate (Fig. 7c–d)
  *Findings*
*LE image* (Fig 7b–c): No specific breast changes are evident.
  Associated features: No
*RC image* (Fig. 7c): No suspicious enhancement
4. *Assessment category*: BI-RADS 1
5. *Management*: The patient was scheduled for a US evaluation after 1 year. The nodule did not display significant changes on US after this period, confirming its benign nature. CEM can again be a problem-solving tool to prevent unnecessary biopsies in patients with benign lesions.

*Tips*: The absence of enhancement on CEM of a breast lesion holds significant predictive value for its benign nature [11]. However, the presence of enhancement does not invariably indicate malignancy. The latest version of BI-RADS incorporates several descriptors enabling the assignment of a suspicion grade to the lesion [11], thereby optimizing biopsy requests for benign lesions. Among the newer descriptors is lesion conspicuity, defined as the enhancement intensity relative to the surrounding parenchyma. According to recent studies [12], high lesion conspicuity is most strongly correlated with lesion malignancy. An intriguing addition to the descriptors is “ground glass,” denoting the ability to visualize the breast parenchyma underlying the lesion enhancement. Unpurified enhancement (lacking the ability to see the underlying parenchyma) appears most associated with lesion malignancy [11]. Lastly, the controversial practice of acquiring late CEM projections (typically 7 min from the start of acquisition) is gaining popularity. Nonetheless, progressively increasing enhancement is often linked with benignity [27].

## Conclusion

CEM is a relatively recent technique that has demonstrated significant advancements in clinical and radiological practice over the past few years, as evidenced by including a dedicated section within the BI-RADS [7]. Our paper seeks to provide a comprehensive overview of the key aspects of the new BI-RADS for CEM and showcase some clinical cases commonly encountered in practice.

A thorough understanding of how breast lesions manifest in CEM can enhance physicians’ awareness of this technique, instilling confidence in its application across various settings and maximizing its potential.

## Data Availability

Not applicable

## Abbreviations

BI-RADS: Breast Imaging Reporting and Data System
CEM: Contrast-enhanced mammography
HE: High energy
LE: Low energy
